# IMPACT OF GENDER ON ASSOCIATION BETWEEN RACE AND DISABILITY: THE CALIFORNIA HEALTH INTERVIEW SURVEY (CHIS)

**DOI:** 10.1093/geroni/igac059.329

**Published:** 2022-12-20

**Authors:** Lien Quach, Christine Vu, Isabelle Tran, Noah Peeri, Uyen-Sa Nguyen

**Affiliations:** Massachusetts General Hospital, Newton, Massachusetts, United States; University of North Texas Health Science Center (UNTHSC), Fort Worth, Texas, United States; Texas Christian University School of Medicine, Fort Worth, Texas, United States; University of North Texas Health Science Center (UNTHSC), Fort Worth, Texas, United States; University of North Texas Health Science Center (UNTHSC), School of Public Health (SPH), Fort Worth, Texas, United States

## Abstract

Women generally have higher prevalence of disability than men in later life. Moreover, Blacks and Hispanics usually have higher prevalence of disability than Whites. Little is known about the impact of gender on the association between race and disability. We used 2015-2016 CHIS data, restricted to adults ≥ 65 years old (n=15,044). Disability was classified as present or absent based on responses on questions related to “to physical, mental, and emotional conditions.” Race was classified as: White, Black, Hispanic, Asian, and Other. We estimated sex- and race-specific proportions and 95% confidence intervals (CI) and used sex-specific multivariable logistic regression to examine the associations between race and disability adjusting for age, education, marital status, cigarette smoking, arthritis, hypertension, and diabetes, mental distress, and walking for work or pleasure. All analyses accounted for complex sampling weights. Approximately 52% of women and 47% of men had disability, while 48% of White, 48% of Black, 60% of Hispanic, 43% of Asian, and 50% of Other race responded as having disability. Adjusting for covariates, Hispanic women had 67% higher odds of having disability compared with White women (OR= 1.67, 95% CI= 1.07–2.60), but there were no differences in male counterparts (OR=1.03, 95% CI=0.68-1.56). Compared with White men, men of Asian or Other race had lower odds for disability, while associations were in the opposite direction in female counterparts; however, associations were not statistically significant. Further research is needed to understand higher prevalence of disability among older minority women compared with White women.

